# Diagnostic accuracy and added value of dual-energy subtraction radiography compared to standard conventional radiography using computed tomography as standard of reference

**DOI:** 10.1371/journal.pone.0174285

**Published:** 2017-03-16

**Authors:** Katharina Martini, Marco Baessler, Stephan Baumueller, Thomas Frauenfelder

**Affiliations:** 1 Institute for Diagnostic and Interventional Radiology, University Hospital Zurich, Zurich, Switzerland; 2 University of Zurich, Zurich, Switzerland; Universidad Francisco de Vitoria, SPAIN

## Abstract

**Purpose:**

To retrospectively evaluate diagnostic performance of dual-energy subtraction radiography (DESR) for interpretation of chest radiographs compared to conventional radiography (CR) using computed tomography (CT) as standard of reference.

**Material and methods:**

A total of 199 patients (75 female, median age 67) were included in this institutional review board (IRB)-approved clinical trial. All patients were scanned in posteroanterior and lateral direction with dual-shot DE-technique. Chest CT was performed within ±72 hours. The system provides three types of images: bone weighted-image, soft tissue weighted-image, herein termed as DESR-images, and a standard image, termed CR-image (marked as CR-image). Images were evaluated by two radiologists for presence of inserted life support lines, pneumothorax, pleural effusion, infectious consolidation, interstitial lung changes, tumor, skeletal alterations, soft tissue alterations, aortic or tracheal calcification and pleural thickening. Inter-observer agreement between readers and diagnostic performance were calculated. McNemar’s test was used to test for significant differences.

**Results:**

Mean inter-observer agreement throughout the investigated parameters was higher in DESR images compared to CR-images (k_DESR_ = 0.935 vs. k_CR_ = 0.858). DESR images provided significantly increased sensitivity compared to CR-images for the detection of infectious consolidations (42% vs. 62%), tumor (46% vs. 57%), interstitial lung changes (69% vs. 87%) and aortic or tracheal calcification (25 vs. 73%) (p<0.05). There were no significant differences in sensitivity for the detection of inserted life support lines, pneumothorax, pleural effusion, skeletal alterations, soft tissue alterations or pleural thickening (p>0.05).

**Conclusion:**

DESR increases significantly the sensibility without affecting the specificity evaluating chest radiographs, with emphasis on the detection of interstitial lung diseases.

## Introduction

Although conventional radiography (CR) of the chest is one of the oldest radiological examinations, CR holds its position in the daily clinical practice due to its broad availability, fast examination time, low cost, and last but not least low radiation dose delivered to the patient [1–4].

However, it is also known that CR has limited diagnostic accuracy in many clinical situations: Previous studies documented lower sensitivity in the detection of pathologic lung changes compared to computed tomography (CT) [5,6]. Heussel et al. [6] reported that in 50% of cases inflammatory pulmonary disease present on CT was not visible in CR. However, thoracic imaging has made significant progress in the last years due to the transition from film-based systems to digital radiography allowing the development of more refined modalities such as dual-energy subtraction radiography (DESR) [7].

This new technique might be able to improve the diagnostic accuracy of CR while maintaining its advantages such as fast examination time, low cost and low radiation dose.

In DESR, additionally to the CR image, a low energy image is acquired. To date two types of dual-energy systems are available: a single-exposure system and a dual-exposure system. Regardless of the used system images are subtracted by a post processing algorithm to produce tissue selective and bone selective images resulting in three different images: 1- bone image, 2- soft tissue image (both marked as DESR images) and 3- standard image (marked as CR-image).

By eliminating bony and calcified structures from the lung parenchyma, DESR may potentially enhance the detection rate of soft-tissue pathologies compared to standard chest X-rays [8], as a recent study reported an improved diagnoistic accuracy by DESR on chest CR for the detection of lung nodules DESR improves diagnostic accuracy of chest CR [7].

Thus, the aim of this study was to provide a comprehensive evaluation of the diagnostic performance of DESR compared to CR for the interpretation of chest radiographs using CT as standard of reference.

## Material and methods

### Patient population

The study was approved by the institutional review board and local ethics committee (KEK Zürich: Cantonal ethics committee Zurich Switzerland). Informed consent was waived because of the retrospective character of this study.

In this observational retrospective study dual-shot-technique radiographs of the chest of 199 in- and outpatients (median age 67.1 years, range 29–93 years; 75 female, 124 male) performed between July 2014 and July 2015 were included. Inclusion criterion was a chest CT performed within 72h of dual-shot technique radiograph. Exclusion criterion was a surgical intervention or manipulation of inserted life support lines (i.e. line placement or removal) between the conventional CR and CT (n = 23).

There is no funding to report for this study.

### Data acquisition

**Fig 1:**
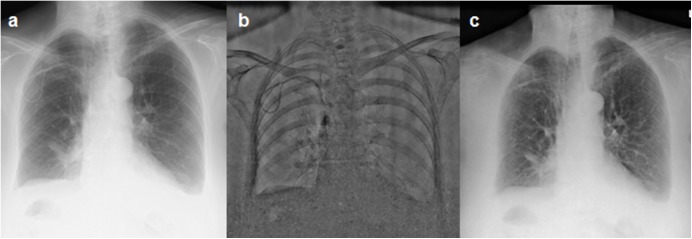
Conventional Radiography and DESR. Conventional radiograph (CR, a). Bone image (b), and soft tissue image (c) of the chest after subtraction of thoracic skeletal structures.

All patients underwent chest radiography in posteroanterior and lateral projection, whereby the posteroanterior projection was obtained with dual-shot DESR-technique (FDR AcSelerate, Fujifilm, Düsseldorf, Germany) at a tube voltage of 130 kVp and a tube current of 7 mA, according to the standard protocol of our department. After a delay of 200 ms the lower energy image (70 kV, 7 mA) was acquired. The higher energy exposure was used to produce the CR image. With the use of a post-processing algorithm the soft tissue and bone image were calculated (**Fig 1**). Radiation dose (Dose Area Products) were recorded for each scan.

Single-energy CT was performed (Somatom Sensation, Somatom Flash and Somatom Force, Siemens Healthcare, Erlangen) with or without intravenously injected contrast agent at 120kVp/110mAs ref and where reconstructed with a slice thickness of 2.0 mm.

### Image analysis

Chest radiographs were evaluated independently by two radiologists (with 18 years and 3 years of experience) for the presence of inserted life support lines (tracheal tubes, central venous catheters, pleural drainages, osteosynthetic material), pneumothorax, mediastinal changes, pleural effusion, infectious consolidation, atelectasis, interstitial lung changes (emphysema, scarring, reticulations and nodular lung changes), lung tumor (masses and solitary nodules), skeletal alterations (fractures, degenerative disorders), aortic or tracheal calcification, and pleural thickening (with and without calcification).

Since DESR has most recently been applied for the evaluation of interstitial lung disease our readers advanced their experience in reading DESR images for the subsequent detection of interstitial lung changes, the first 20 patients (according to the scan date) were declared as “learning cases”, by which readers got acquainted to alterations in the appearance of interstitial lung parenchyma in DESR comparing the images to the respective CT studies. Consequently, remaining 179 patients were implemented into statistical analysis of interstitial lung changes.

Additionally the images were evaluated regarding the presence of motion artifacts occurring between the two shots (yes / no). Reading results such as findings and the location of findings within the lobes were recorded in premade tables. For the CR-image interpretation only CR images were evaluated by the readers. For the DESR-image interpretation, the CR images along with the bone- and the soft tissue image were evaluated and the. Both readers were blinded for the clinical indication of the radiographies.

A total of 398 data sets were reviewed (2 data sets per patient). Any recall bias was ruled out by evaluating the correspondent CR and DESR image by at least one month time lapsed in between diagnostic sessions.

Chest CT served as the standard of reference. CT images were evaluated in consensus by both readers two weeks after assessing the radiographs.

Evaluation of CR images, DESR images, and CT examinations was performed using the picture archiving and communications system (PACS) with a high definition liquid crystal display monitor (BARCO; Medical Imaging System, Kortrijk, Belgium) using Impax (Version 6.4.0.455; Agfa-Gevaert, Mortsel, Belgium).

### Statistical analysis

Continuous variables were expressed as means ± standard deviation (SD) while categorical variables were expressed as frequencies or percentages. Inter-observer agreement between readers was assessed using Cohen’s Kappa (κ) for each pair of variables. According to Landis and Koch, kappa-values of 0.61 to 0.80 were interpreted as substantial while values between 0.81 and 1.00 were interpreted as excellent agreement [9]. Furthermore, specificity and sensitivity were calculated for detection of lung pathologies. McNemar’s test was used to test for significant differences. Since multiple comparisons were performed a Bonferroni-corrected two-tailed p-value of p < 0.004 was considered as statistical significant.

Statistical analysis was performed using commercially available software (SPSS, release 22.0; SPSS, Chicago, IL, USA).

## Results

### Patient population

**Fig 2:**
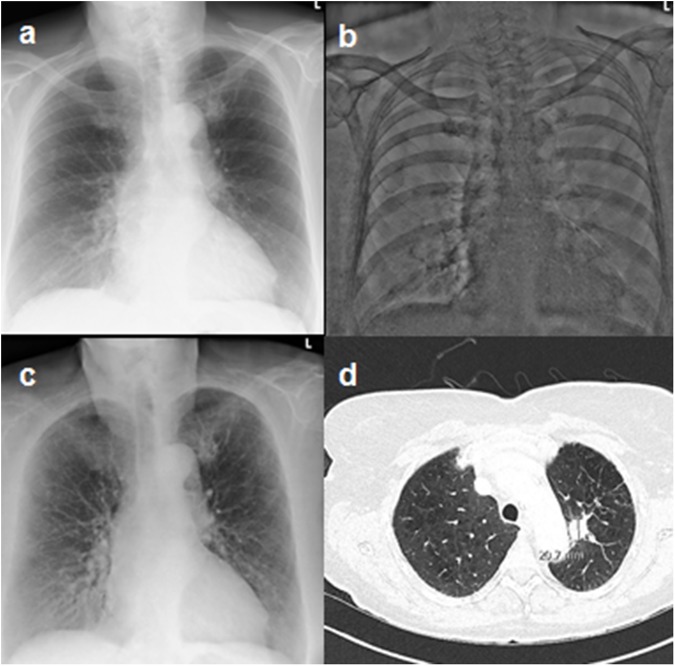
Lung mass on different imaging modalities. 61 year old female patient showing a focal increase in density in the left upper lobe in the CR image (a). This focal increase in density is not visible on the bone image (b) while it is clearly depicted by the soft tissue image (c) and therefore is primarily suspicious for a soft tissue mass in the left upper lobe. CT (d) confirms the presence of a soft tissue mass in the left upper lobe adjacent to the aortic arch suspicious for a bronchus carcinoma.

The majority of patients included in our study were referred to our department for the evaluation of cardiovascular compensation (n = 70) followed by preoperative imaging (n = 55). Other clinical questions comprised evaluation of the presence of lung consolidations (n = 44), pneumothorax (n = 26) or lung masses (n = 4) (**Table 1, Fig 2, S1 File**). The mean time interval between the acquisition of the conventional images and CT was of 28.7 hours (min 1 hour; max 70 hours).

**Table 1:** Patient characteristics.

		Total(n = 199)
***Female: Male***		75: 125
***Median age (y)***		67 (29–93)
***Clinical question***	Preoperative imaging	55
	Infective consolidation	44
	Cardio-vascular situation	70
	Lung mass	4
	Pneumothorax	26

Number of cases (n), years (y).

### Radiation dose

Compared to CR, DESR required higher radiation doses. However, mean dose area product (DAP) for DESR was 16% higher than that of CR. The mean DAP for DESR was 1613 Gycm2 and for CR image alone was 1397 Gycm2

### Image analysis

Mean inter-observer agreement throughout the investigated parameters was higher in DESR images compared to CR images (κ_DESR_ = 0.935 vs. κ_CR_ = 0.858) (**Table 2, S1 File**).

**Table 2:** Inter-reader agreement in detection of pathologic lung changes and inserted life support lines.

Lung Changes	CRReader 1 vs. 2	DESRReader 1 vs. 2
*Inserted life support lines*	1.000	1.000
*Pneumothorax*	1.000	1.000
*Mediastinal changes*	0.802	0.864
*Pleural effusion*	0.989	0.989
*Infective consolidations*	0.922	0.964
*Lung atelectasis*	0.852	0.967
*Lung masses*	0.767	0.911
*Skeletal alterations*	0.814	0.814
*Soft tissue alterations*	0.942	0.969
*Calcification (aorta/trachea)*	0.886	0.963
*Pleural thickening*	0.923	0.784
*Emphysema*	0.958	0.957
*Reticular changes*	0.962	1.000
*Nodular changes*	1.000	1.000
*Scarring*	0.985	1.000

Conventional Radiography (CR), Dual-energy subtraction radiography (DESR).

DESR images showed tendencies to improve sensitivity for the detection of infectious consolidations (40% vs. 60%; p = 0.022) as well as of aortic or tracheal calcification (23% vs. 71%; p = 0.008) compared to CR images, however differences were not statistical significant. DESR surpassed the diagnostic sensitivity provided by CR for the detection interstitial lung changes (69% vs. 87%; p < 0.004).

**Fig 3:**
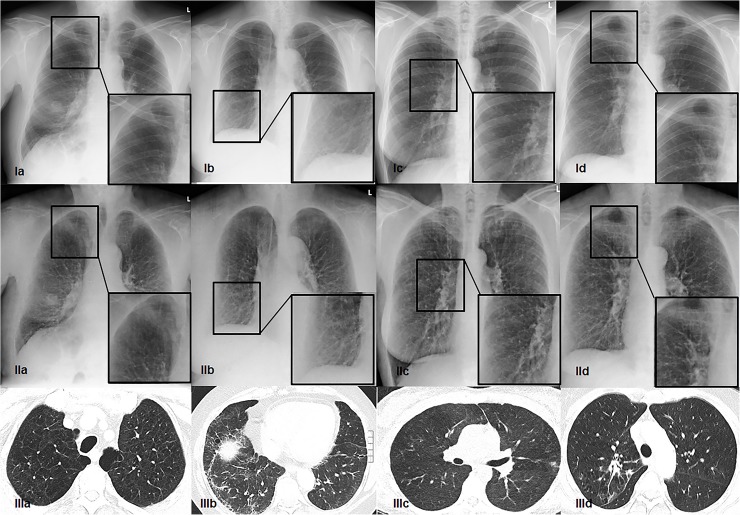
Interstitial lung changes in different imaging modalities. Appearance of interstitial lung changes such as (a) emphysema, (b) reticular lung changes, (c) nodular lung changes and (d) scarring of lung parenchyma with (I) CR, (II) DESR soft tissue image and (III) CT in four different patients. While nodular changes are visible in all the three imaging modalities (Ic, IIc and IIIc), emphysema, reticular lung changes and lung scarring are better depicted in the DESR soft tissue image (IIa, IIb and IId) compared to the CR image (Ia, Ib and Id). The patient having the emphysema had also a solid lung mass in the right lower lobe (Ia and IIa).

DESR further showed a significantly enhanced sensitivity for the detection of lung emphysema (75% vs. 44%) as well as for the detection of scarring (96% vs. 81%) (p < 0.004). While there was no significant difference in the specificity for the detection of emphysema, reticular lung changes and scarring (**Table 3, Fig 3, S1 File**), the specificity for the detection of lung scarring was significantly higher using CR compared to DESR (76% vs. 69%, p < 0,05). There were only two cases of nodular lung changes detected by CT that were visible in both DESR and CR images (**Table 3, S1 File**).

**Table 3:** Sensitivity and specificity in detection of interstitial lung changes in detail.

Lung Changes	Sensitivity %		p-Value	Specificity %			p-Value
	CR(R1/R2)	DESR(R1/R2)	CR vs. DESR	CR(R1/R2)		DESR(R1/R2)	CR vs. DESR
*Lung emphysema*	44 / 46	75 / 76	p < 0.001	85 / 84		84 / 83	p > 0.004
*Scarring*	81 / 74	96 / 96	p = 0.002	76 / 76		69 / 70	p < 0.004
*Reticular lung changes*	77 / 85	92 / 92	p = 0.500	87 / 87		86 / 86	p > 0.004
Nodular lung changes*	-- / --	-- / --	----	-- / --		-- / --	----

Conventional Radiography (CR), Dual-energy subtraction radiography (DESR)

* only two case showed nodular lung changes on CT, therefore no statistical calculations could be performed.

There were no significant differences seen in sensitivity for the detection of inserted life support lines, pneumothorax, pleural effusion, skeletal alterations, soft tissue alterations or pleural thickening (all p > 0.05).

Unfortunately, only four out of 199 patients showed the presence of lung masses, and therefore statistical analyses could not be performed.

Specificity was high regardless which type of radiography acquisition technique was applied (**Table 4, Fig 2, S1 File**). In the 398 evaluated images both readers did not report any motion artifacts.

**Table 4:** Sensitivity and Specificity in detection of pathologic lung changes and inserted life support lines.

Lung Changes	Sensitivity %		p-Value	Specificity %		p-Value
	CR(R1/R2)	DESR(R1/R2)	CR vs. DESR	CR(R1/R2)	DESR(R1/R2)	CR vs. DESR
*Inserted life support lines*	91 / 91	91 / 91	1.000	100 / 100	100 / 100	p > 0.004
*Pneumothorax*	58 / 58	58 / 58	1.000	100 / 100	99 / 99	p > 0.004
*Mediastinal changes*	53 / 82	59 / 82	1.000	99 / 99	98 / 98	p > 0.004
*Pleural effusion*	67 / 68	69 / 68	1.000	98 / 98	95 / 95	p > 0.004
*Infective consolidations*	40 / 42	60 / 62	0.022	99 / 97	97 / 96	p > 0.004
*Lung atelectasis*	22 / 22	65 / 69	0.001	99 / 98	99 / 99	p > 0.004
Lung tumor*	-- / --	-- / --	-- / --	-- / --	-- / --	-- / --
*Skeletal alterations*	42 / 52	42 /50	1.000	100 / 100	100 / 100	p > 0.004
*Soft tissue alterations*	82 / 73	73 / 73	1.000	99 / 99	99 / 99	p > 0.004
*Calcification (aorta/trachea)*	24 / 29	71 / 76	0.008	100 / 100	99 / 99	p > 0.004
*Pleural thickening*	70 /70	70 / 80	0.625	100 / 100	100 / 100	p > 0.004

Conventional Radiography (CR), Dual-energy subtraction radiography (DESR)

* only 4 cases, therefore statistical analysis could not be performed.

## Discussion

Our study showed that the use of DESR compared to CR for chest diagnostics increased the sensitivity as well as the inter-reader agreement for the detection of many pathologic lung changes while there was no significant change in specificity between the two imaging modalities.

CR of the chest is still the most frequently performed radiological examination in daily clinical routine. CR has a high availability, low cost, and low radiation dose delivered to the patient [1–4], while suffering some limitations such as a moderate diagnostic accuracy for the detection of pathologic lung changes compared to other imaging modalities such as CT [5,6].

CR is a projection-based imaging method, i.e. a three-dimensional structure is projected onto a two-dimensional image. Therefore, despite the high spatial resolution, it often lacks the possibility to differentiate structures with equal density adjacent to each other or suffers from superposition of different structures [10]. Thus, the assessment of small density increase in the lungs is often hampered and lung lesions located behind bony structures might be often missed compared to CT.

To date two types of dual-energy systems are available: a single-exposure system and a dual-exposure system. In this work we used the dual-exposure system which has a higher signal to noise ratio compared to the single exposure technique but is also known to be more sensitive to motion artifacts [7]. In the single exposure system two phosphor plates separated by a copper filter are exposed to the X-ray beam. While the front plate receives the unfractionated beam and produces a standard chest radiograph, the back plate receives the high energy photons not selected out by the front plate and the copper filter [7]. In the dual-exposure system two images are acquired with a delay of 200 ms, one at 70 kV and one at 130 kV. Slight patient movement, breathing, and also pulsation of the heart between the two acquisitions can result in mis-registration artifacts on the subtracted image [11]. However, in our study we did not experience this type of motion artifacts in all evaluated images.

Different body tissues have different attenuation properties at different tube voltages due to different attenuation coefficients. DESR uses these differences in attenuation to generate tissue selective images [11]. A post-processing algorithm enables the subtraction of structures that contain calcium (i.e. bone) from the image resulting in an image displaying only soft tissue and lung parenchyma, the so called “soft tissue DESR image” [12].

DESR of the chest can improve the visualization of lung alterations (i.e. lung nodules or interstitial lung changes) by subtracting overlying bone structures such as the rips, the spine or the shoulder girdle. Lung nodules or tumors located behind bony structures and therefore not being visible on standard CR images become visible on the soft tissue DESR image as they are no longer superimposed by the above mentioned skeletal structures.

Some studies showed that DESR improves diagnostic accuracy of chest radiographs [7]. However, most of these studies focused mainly on the detection of lung nodules [7, 13, 14], investigated a rather limited number of patients [7, 13, 14]or had a rather long period between the acquisition of the DESR images and the reference [7]. Li *et al*. who evaluated the accuracy and confidence of radiologists in diagnosing pulmonary nodules on 19 previously missed lung cancer nodules showed an increase of both factors [14]. Uemura *et al*. [13] reported similar results showing an increase in diagnostic accuracy in the reading of DESR images of 52 patients with pulmonary nodules as compared to CR images [13]. The bone selective image helps to evaluate if structures seen on CR are calcified or not and therefore allow for discrimination of harmless calcified granulomas from possible malignant soft tissue lung masses. In this study we did not differentiate between the detection of calcified nodules or non-calcified nodules but we could show that DESR has a significantly higher detection rate of aortic and tracheal calcification compared to CR.

Due to the relative novelty of DESR it has yet to find its niche in radiology [7]. Some authors claim a potential application for DESR in chest imaging for screening purposes in patients at high risk of lung cancer such as for example heavy smokers or patients with occupational exposures. However our results do not support this usage due to the moderate sensitivity for the detection of small lung nodules. Another potential field of application might be the detection and evaluation of lung emphysema, scarred lung parenchymal changes, and lung reticulations regarding interstitial lung fibrosis since we found a significantly increased and very high sensitivity for the detection of such pulmonary pathologies. In our study DESR the greatest impact on the diagnosis of infectious and interstitial lung diseases by increasing the sensitivity and interreader agreement. Therefore, DESR appears to be a promising alternative to CR ruling out pulmonary infection with high diagnostic accuracy, such as for example in immunocompromised patients who are repetitively screened for pneumonia. The high sensitivity for the detection of pneumothorax, pulmonary consolidations and osseous lesions make DESR a valuable tool keeping current strength of CR as a fast, widely available, and easy to perform imaging modality at low cost and low radiation dose while reducing current drawback regarding diagnostic sensitivity [5,6].

Although DESR has to compete with the rising numbers of low-dose CT protocols providing high diagnostic accuracy while radiation dose is close to that of a CR examination [15], the high sensitivity for the above mentioned findings establishes this modality as a valuable alternative in emergency settings, where fast diagnosis and patient throughput are important factors. We estimate that DESR could also give additional value of clinical significance where unclear findings on conventional radiography can be cleared with soft tissue and bone reconstructions of DESR and make further CT follow up redundant resulting in lower radiation dose delivered to the patient. Additionally, DESR as an imaging technique being fast and easy to perform, combined with lower costs compared to CT could attain much higher sensitivity of the detection of pathologic changes, especially in settings deprived of access to CT.

Compared to CR DESR required higher radiation doses. However, since DESR images were only obtained for posteroanterior projections and not for lateral projections the overall increase of radiation dose did not exceed 16% in our study. MacMahon et al. [16] propose a method to perform DESR without increasing radiation dose by reducing the radiation dose of the lateral image for the value which was used to acquire the additional posteroanterior image. Although a direct comparison to dosage used in ultra-low-dose CT protocols is not possible our results show that DESR is still on a lower level. Our study has some limitations: First, since the time interval between the acquisition of the chest radiograph and the chest CT was up to 72 hours, some conditions such as infectious consolidations might have changed. Second, only patients who obtained additional CT examination next to the chest radiography were eligible for the study. This could favor the inclusion of patients with a high pretest probability of disease and therefore might result in a selection bias. Third, we did not differentiate between solid and sub-solid pulmonary nodules due to substantial inter-observer variability for nodule classification on low radiation dose scans as previously shown [17].

In conclusion, DESR increases significantly the sensibility with no change in specificity in the evaluation of chest radiographs, especially concerning detection of interstitial lung diseases.

## Supporting information

S1 File Data supporting information file. Statistical considerations in detail.
(DOCX)
