# Supplementary material for: Diagnostic accuracy and added value of dual-energy subtraction radiography compared to standard conventional radiography using computed tomography as standard of reference
Source: PLoS One. 2017 Mar 16;12(3):e0174285. doi: 10.1371/journal.pone.0174285 (PMC5354458; doi:10.1371/journal.pone.0174285)

**Data supporting information file**

| **Patient Characteristics** | | |
| --- | --- | --- |
|  |  | **Total**  **(n=199)** |
| ***Female : Male*** |  | 75 : 125 |
| ***Median age (y)*** |  | 67 |
| ***Range of age (y)*** |  | 29-93 |
| ***Clinical question*** | Preoperative imaging | 55 |
|  | Infective consolidation | 44 |
|  | Cardio-vascular situation | 70 |
|  | Lung mass | 4 |
|  | Pneumothorax | 26 |
|  | | |
| Number of cases (n), years (y). | | |

| **Pathologic lung changes and inserted life support lines** | |
| --- | --- |
|  | **(n)** |
| *Inserted life support lines* | 80 |
| *Pneumothorax* | 19 |
| *Mediastinal changes* | 17 |
| *Pleural effusion* | 100 |
| *Infective consolidations* | 45 |
| *Lung atelectasis* | 23 |
| *Lung masses* | 4 |
| *Skeletal alterations* | 31 |
| *Soft tissue alterations* | 22 |
| *Calcification (aorta/trachea)* | 17 |
| *Pleural thickening* | 20 |
| *Emphysema** | 59 |
| *Reticular changes** | 13 |
| *Nodular changes** | 2 |
| *Scarring** | 47 |
|  | |
| Number of cases (n). Only 179 out of 199 cases evaluated (learning cases (n=20) excluded from statistical considerations | |

| **Pathologic lung changes and inserted life support lines in learning cases** | |  |
| --- | --- | --- |
|  | **(n)** |  |
| *Emphysema* | 4 |  |
| *Reticular changes* | 2 |  |
| *Nodular changes* | 0 |  |
| *Scarring* | 7 |  |
|  | |  |
| Number of cases (n). First scanned patients according to scan date | |  |
| **Inter-reader agreement in detection of pathologic lung changes and inserted life support lines**  *Inserted life support lines*  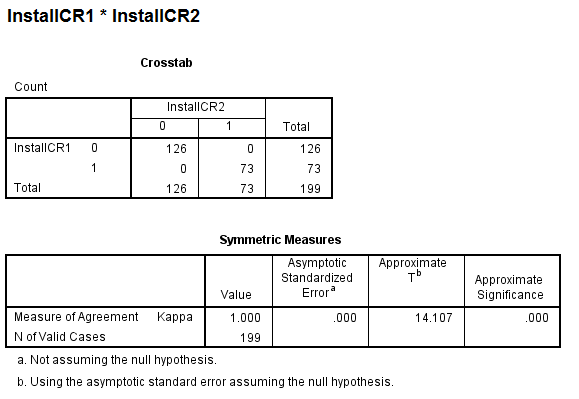  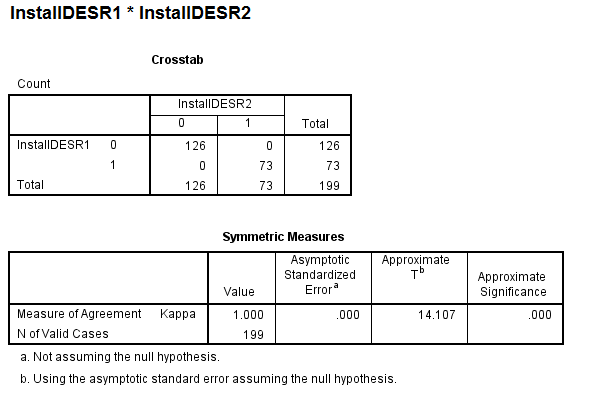  *Pneumothorax*  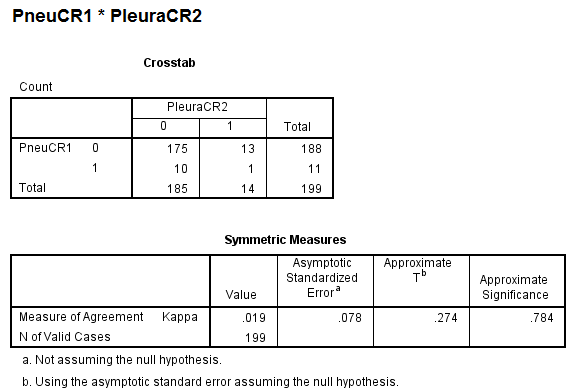  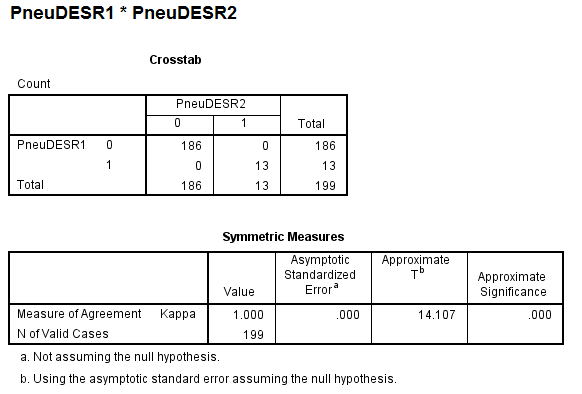  *Mediastinal changes*  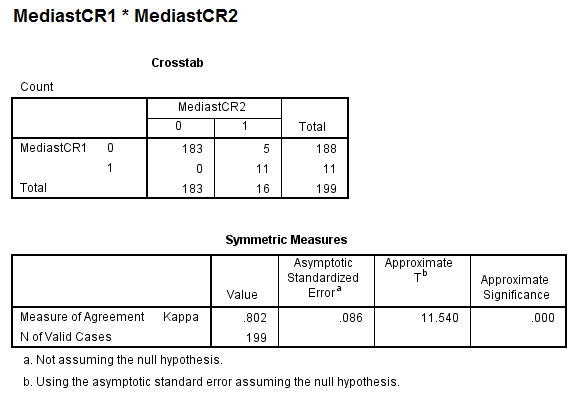  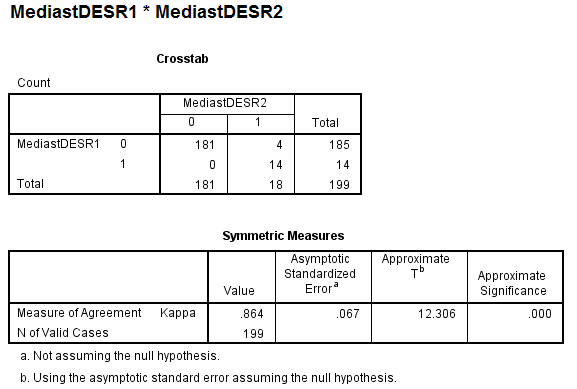  *Pleural effusion:*  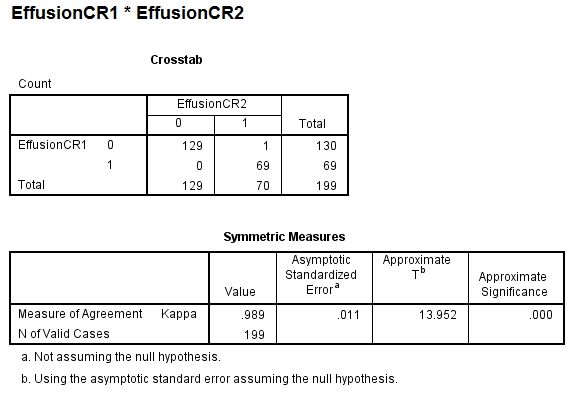  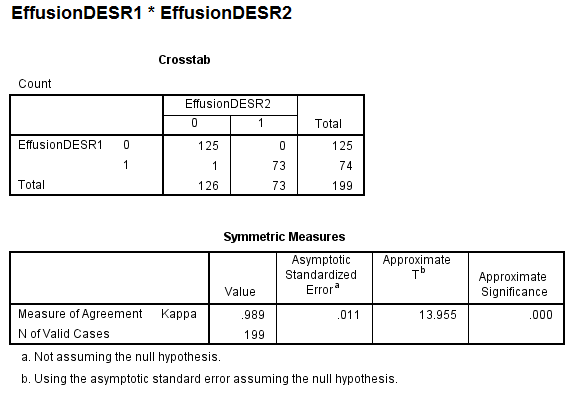  *Consolidations:*  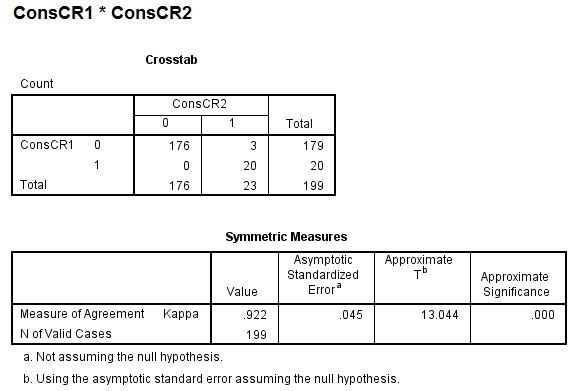  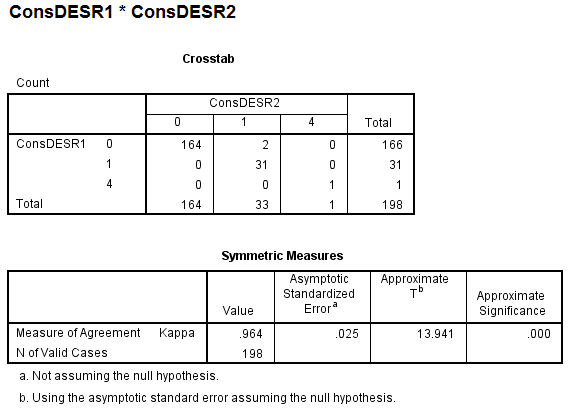  *Lung atelectasis*  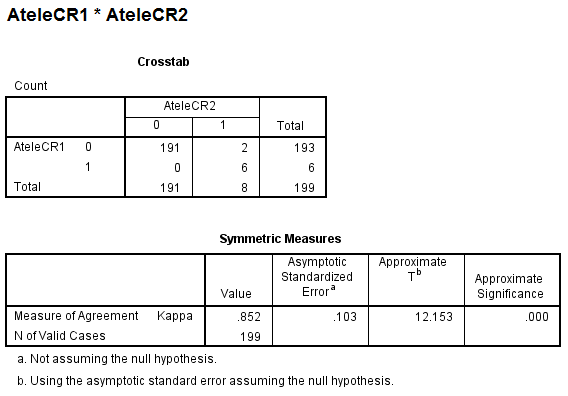  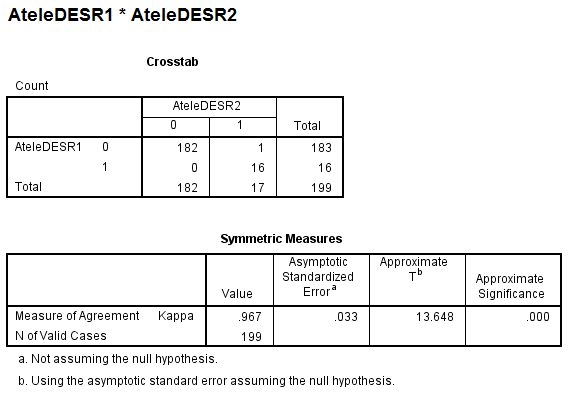  *Lung masses*  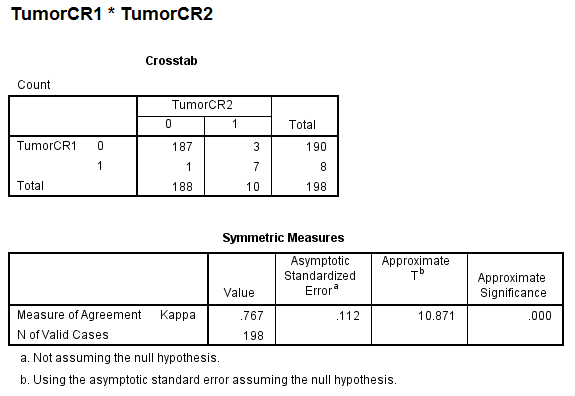  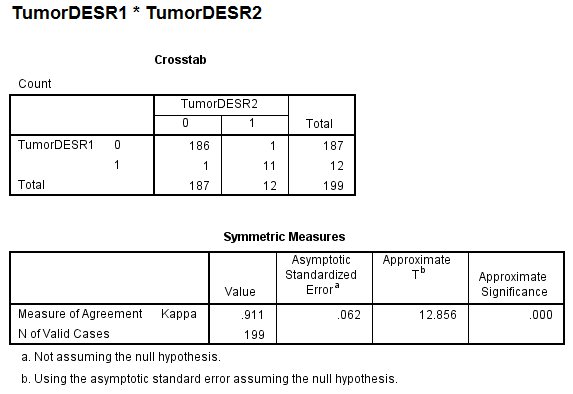  *Skeletal alterations*  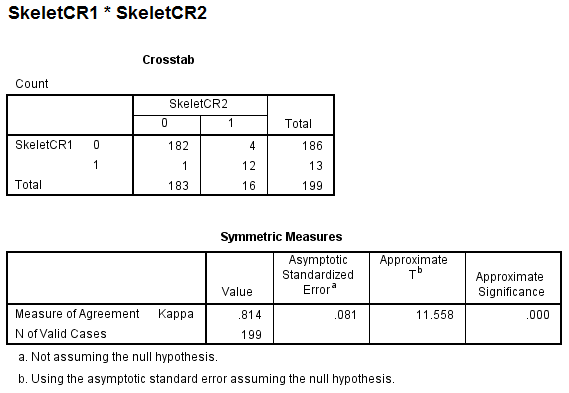  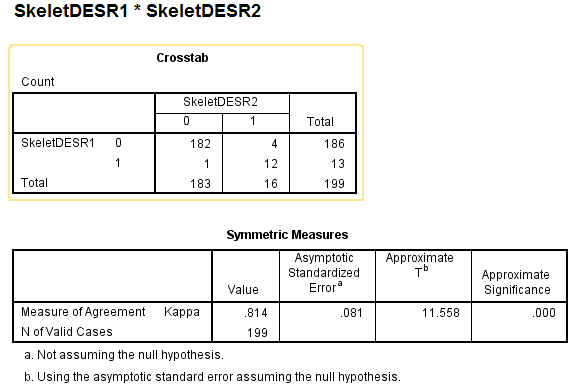  *Soft tissue alterations*  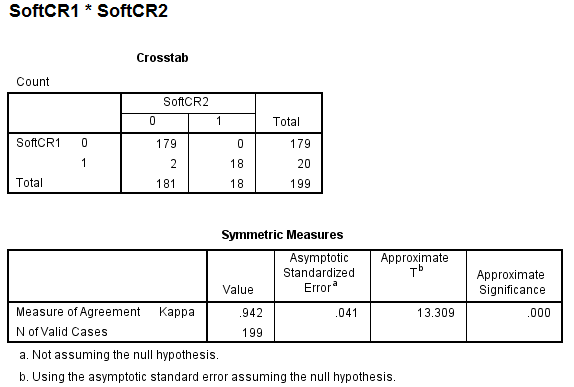  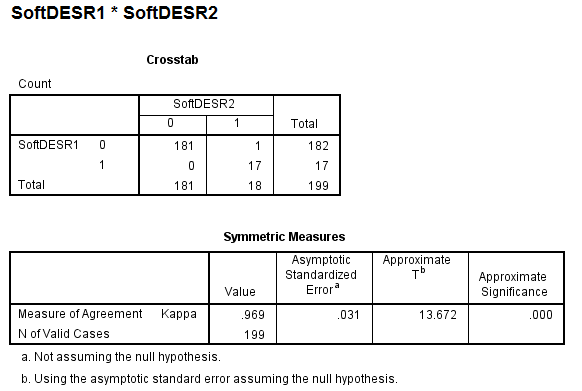  *Calcification*  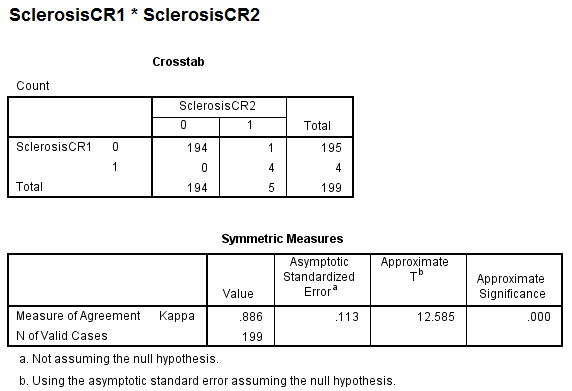  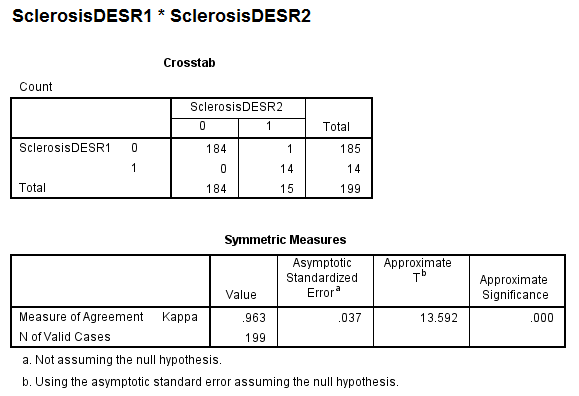  *Pleural thickening*  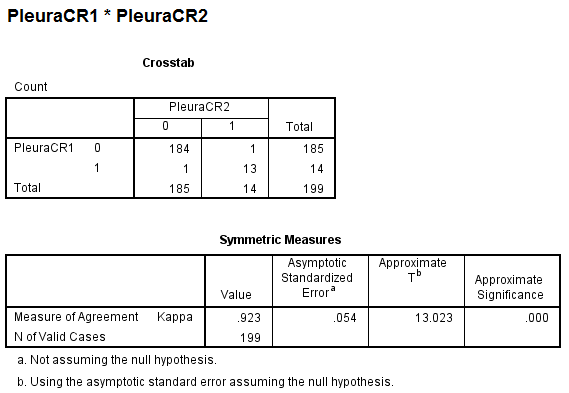  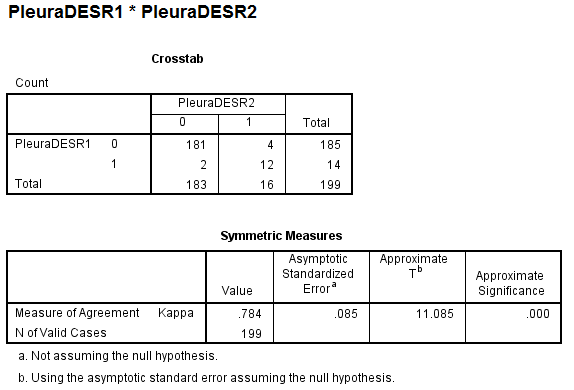  *Emphysema*  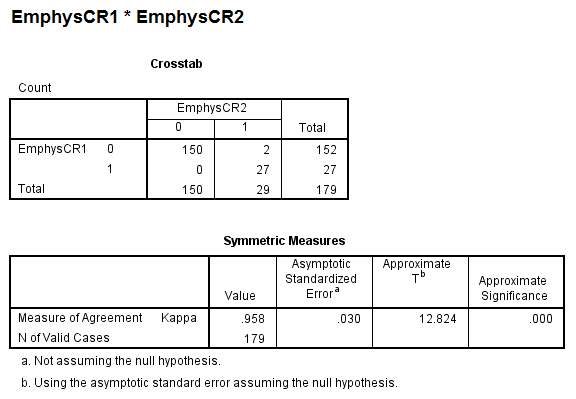  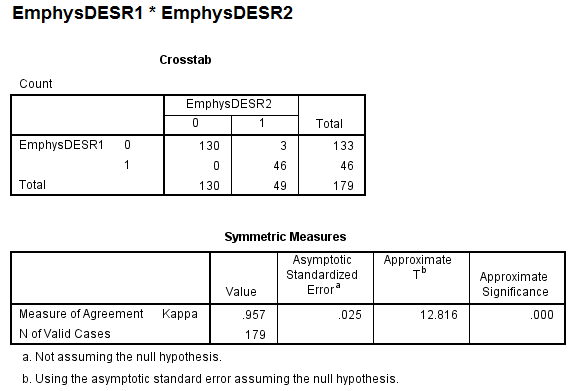  *Reticular lung changes*  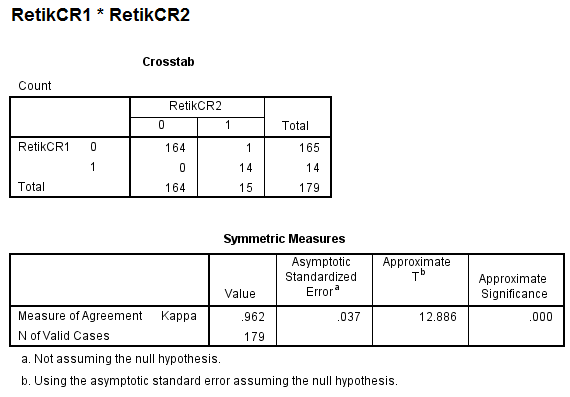  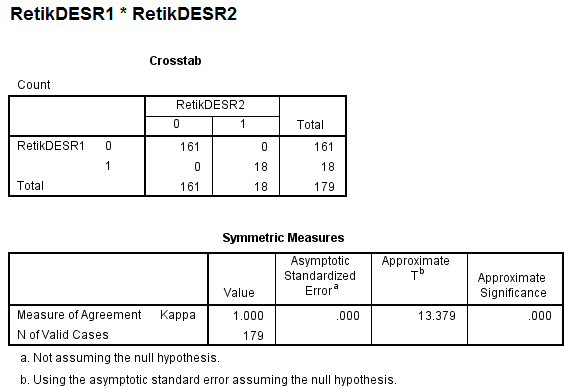  *Nodular lung changes*  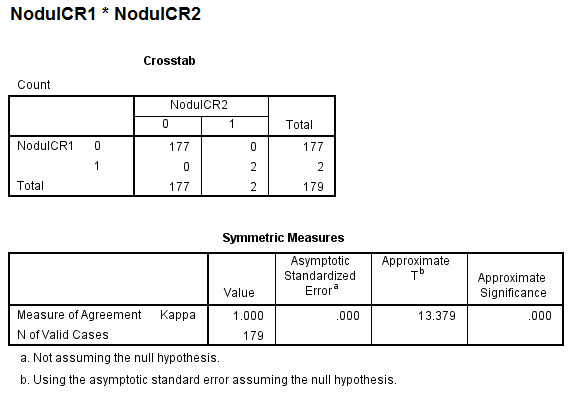  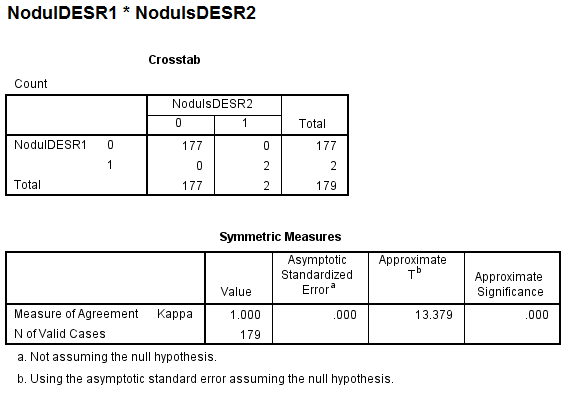  *Scarring* | | |


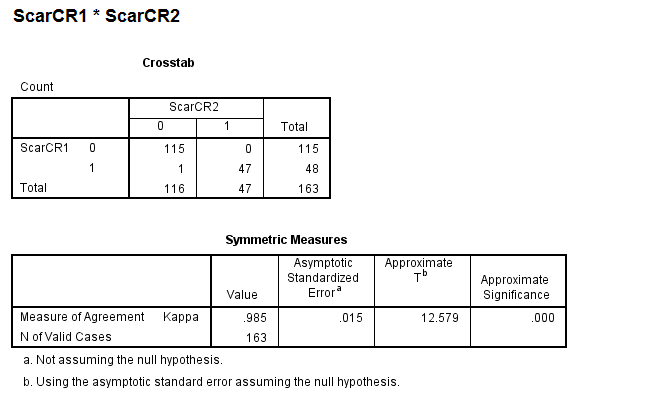


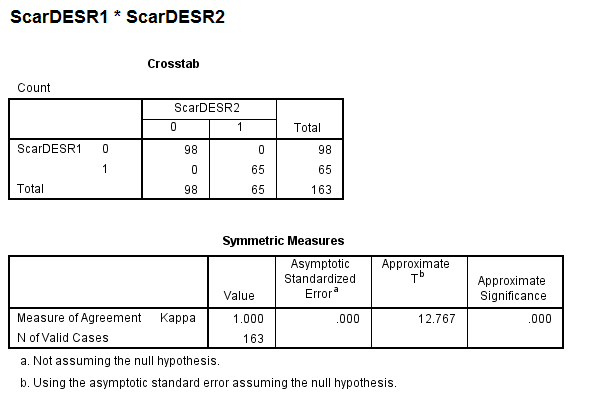


**Sensitivity and Specificity in detection of pathologic lung changes and inserted life support lines**

| *Inserted life support lines*   \|  \| ***CR_R1*** \| ***CR_R2*** \| ***DESR_R1*** \| ***DESR_R2*** \| \| --- \| --- \| --- \| --- \| --- \| \|  \|  \|  \|  \|  \| \| ***Sensitivity*** \| *0,9125* \| *0,9125* \| *0,9125* \| *0,9125* \| \|  \|  \|  \|  \|  \| \| ***Specificity*** \| *1* \| *1* \| *1* \| *1* \| |
| --- | --- | --- | --- | --- | --- | --- | --- | --- | --- | --- | --- | --- | --- | --- | --- | --- | --- | --- | --- | --- | --- | --- | --- | --- | --- |
| *Pneumothorax*   \|  \| ***CR_R1*** \| ***CR_R2*** \| ***DESR_R1*** \| ***DESR_R2*** \| \| --- \| --- \| --- \| --- \| --- \| \|  \|  \|  \|  \|  \| \| ***Sensitivity*** \| *0,5789* \| *0,5789* \| *0,5789* \| *0,5789* \| \|  \|  \|  \|  \|  \| \| ***Specificity*** \| *1* \| *1* \| *0,9889* \| *0,9889* \| |
| *Mediastinal changes*   \|  \| ***CR_R1*** \| ***CR_R2*** \| ***DESR_R1*** \| ***DESR_R2*** \| \| --- \| --- \| --- \| --- \| --- \| \|  \|  \|  \|  \|  \| \| ***Sensitivity*** \| *0,5294* \| *0,8235* \| *0,5882* \| *0,8235* \| \|  \|  \|  \|  \|  \| \| ***Specificity*** \| *0,989* \| *0,989* \| *0,978* \| *0,978* \| |
| *Pleural effusion*   \|  \| ***CR_R1*** \| ***CR_R2*** \| ***DESR_R1*** \| ***DESR_R2*** \| \| --- \| --- \| --- \| --- \| --- \| \|  \|  \|  \|  \|  \| \| ***Sensitivity*** \| *0,67* \| *0,68* \| *0,69* \| *0,68* \| \|  \|  \|  \|  \|  \| \| ***Specificity*** \| *0,9798* \| *0,9798* \| *0,9495* \| *0,9495* \| |
| *Infective consolidations*   \|  \| ***CR_R1*** \| ***CR_R2*** \| ***DESR_R1*** \| ***DESR_R2*** \| \| --- \| --- \| --- \| --- \| --- \| \|  \|  \|  \|  \|  \| \| ***Sensitivity*** \| *0,4* \| *0,4222* \| *0,6* \| *0,6222* \| \|  \|  \|  \|  \|  \| \| ***Specificity*** \| *0,987* \| *0,974* \| *0,9675* \| *0,961* \| |
| *Lung atelectasis*   \|  \| ***CR_R1*** \| ***CR_R2*** \| ***DESR_R1*** \| ***DESR_R2*** \| \| --- \| --- \| --- \| --- \| --- \| \|  \|  \|  \|  \|  \| \| ***Sensitivity*** \| *0,2174* \| *0,2174* \| *0,6522* \| *0,6956* \| \|  \|  \|  \|  \|  \| \| ***Specificity*** \| *0,9943* \| *0,983* \| *0,9943* \| *0,9943* \| |
| *Lung tumor**   \|  \| ***CR_R1*** \| ***CR_R2*** \| ***DESR_R1*** \| ***DESR_R2*** \| \| --- \| --- \| --- \| --- \| --- \| \|  \|  \|  \|  \|  \| \| ***Sensitivity*** \| *0,5* \| *0,75* \| *0,75* \| *0,75* \| \|  \|  \|  \|  \|  \| \| ***Specificity*** \| *0,9692* \| *0,959* \| *0,9538* \| *0,9538* \| |
| *Skeletal alterations*   \|  \| ***CR_R1*** \| ***CR_R2*** \| ***DESR_R1*** \| ***DESR_R2*** \| \| --- \| --- \| --- \| --- \| --- \| \|  \|  \|  \|  \|  \| \| ***Sensitivity*** \| *0,4194* \| *0,5161* \| *0,4194* \| *0,5161* \| \|  \|  \|  \|  \|  \| \| ***Specificity*** \| *1* \| *1* \| *1* \| *1* \| |
| *Soft tissue alterations*   \|  \| ***CR_R1*** \| ***CR_R2*** \| ***DESR_R1*** \| ***DESR_R2*** \| \| --- \| --- \| --- \| --- \| --- \| \|  \|  \|  \|  \|  \| \| ***Sensitivity*** \| *0,8182* \| *0,7273* \| *0,7273* \| *0,7273* \| \|  \|  \|  \|  \|  \| \| ***Specificity*** \| *0,9887* \| *0,9887* \| *0,9887* \| *0,9887* \| |
| *Calcification (aorta/trachea)*   \|  \| ***CR_R1*** \| ***CR_R2*** \| ***DESR_R1*** \| ***DESR_R2*** \| \| --- \| --- \| --- \| --- \| --- \| \|  \|  \|  \|  \|  \| \| ***Sensitivity*** \| *0,2353* \| *0,2941* \| *0,7059* \| *0,7647* \| \|  \|  \|  \|  \|  \| \| ***Specificity*** \| *1* \| *1* \| *0,989* \| *0,989* \| |
| *Pleural thickening*   \|  \| ***CR_R1*** \| ***CR_R2*** \| ***DESR_R1*** \| ***DESR_R2*** \| \| --- \| --- \| --- \| --- \| --- \| \|  \|  \|  \|  \|  \| \| ***Sensitivity*** \| *0,7* \| *0,7* \| *0,7* \| *0,8* \| \|  \|  \|  \|  \|  \| \| ***Specificity*** \| *1* \| *1* \| *1* \| *1* \| |
| *Emphysema*   \|  \| ***CR_R1*** \| ***CR_R2*** \| ***DESR_R1*** \| ***DESR_R2*** \| \| --- \| --- \| --- \| --- \| --- \| \|  \|  \|  \|  \|  \| \| ***Sensitivity*** \| *0,4407* \| *0,4576* \| *0,7458* \| *0,7627* \| \|  \|  \|  \|  \|  \| \| ***Specificity*** \| *0,85* \| *0,8429* \| *0,8429* \| *0,8286* \| |
| *Reticular changes*   \|  \| ***CR_R1*** \| ***CR_R2*** \| ***DESR_R1*** \| ***DESR_R2*** \| \| --- \| --- \| --- \| --- \| --- \| \|  \|  \|  \|  \|  \| \| ***Sensitivity*** \| *0,7692* \| *0,8462* \| *0,9231* \| *0,9231* \| \|  \|  \|  \|  \|  \| \| ***Specificity*** \| *0,871* \| *0,871* \| *0,8602* \| *0,8602* \| |
| *Nodular changes*   \|  \| ***CR_R1*** \| ***CR_R2*** \| ***DESR_R1*** \| ***DESR_R2*** \| \| --- \| --- \| --- \| --- \| --- \| \|  \|  \|  \|  \|  \| \| ***Sensitivity*** \| *1* \| *1* \| *1* \| *1* \| \|  \|  \|  \|  \|  \| \| ***Specificity*** \| *0,8985* \| *0,8985* \| *0,8985* \| *0,8985* \| |
| *Scarring*   \|  \| ***CR_R1*** \| ***CR_R2*** \| ***DESR_R1*** \| ***DESR_R2*** \| \| --- \| --- \| --- \| --- \| --- \| \|  \|  \|  \|  \|  \| \| ***Sensitivity*** \| *0,8085* \| *0,7447* \| *0,9574* \| *0,9574* \| \|  \|  \|  \|  \|  \| \| ***Specificity*** \| *0,7632* \| *0,7632* \| *0,6908* \| *0,6974* \| |

Conventional Radiography (CR), Dual-energy subtraction radiography (DESR), Reader 1 (R1), Reader 2 (R2) * only 4 cases, therefore statistical analysis could not be performed.

**P-values with McNemar for Sensitivity and Specificity in detection of pathologic lung changes and inserted life support lines**


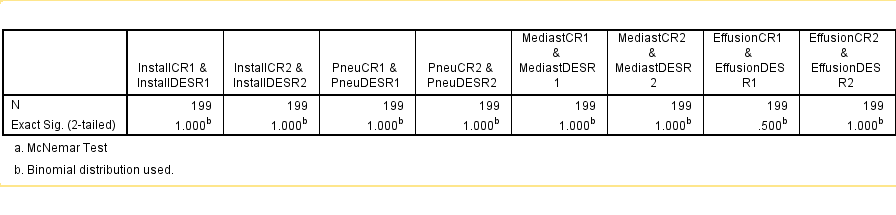


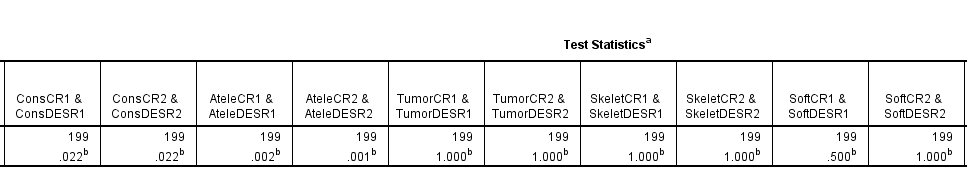


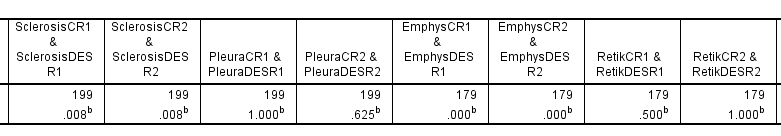


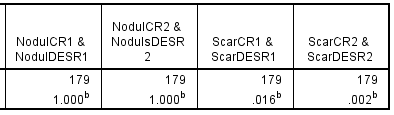

Supplement: S1 File — Statistical considerations in detail. (DOCX) [file pone.0174285.s001.docx]
